# Diagnostic challenges and management advances in cytochrome P450 oxidoreductase deficiency, a rare form of congenital adrenal hyperplasia, with 46, XX karyotype

**DOI:** 10.3389/fendo.2023.1226387

**Published:** 2023-08-11

**Authors:** Chunqing Wang, Qinjie Tian

**Affiliations:** ^1^Department of Ultrasound, Beijing Tiantan Hospital, Capital Medical University, Beijing, China; ^2^Department of Obstetrics and Gynecology, Peking Union Medical College Hospital, Chinese Academy of Medical Sciences, Beijing, China

**Keywords:** cytochrome P450 oxidoreductase deficiency, congenital adrenal hyperplasia, ovarian cyst, adrenal development, menstrual disorders, disorders of sex development, skeletal malformations

## Abstract

Cytochrome P450 oxidoreductase deficiency (PORD) is a rare form of congenital adrenal hyperplasia that can manifest with skeletal malformations, ambiguous genitalia, and menstrual disorders caused by cytochrome P450 oxidoreductase (POR) mutations affecting electron transfer to all microsomal cytochrome P450 and some non-P450 enzymes involved in cholesterol, sterol, and drug metabolism. With the advancement of molecular biology and medical genetics, increasing numbers of PORD cases were reported, and the clinical spectrum of PORD was extended with studies on underlying mechanisms of phenotype–genotype correlations and optimum treatment. However, diagnostic challenges and management dilemma still exists because of unawareness of the condition, the overlapping manifestations with other disorders, and no clear guidelines for treatment. Delayed diagnosis and management may result in improper sex assignment, loss of reproductive capacity because of surgical removal of ruptured ovarian macro-cysts, and life-threatening conditions such as airway obstruction and adrenal crisis. The clinical outcomes and prognosis, which are influenced by specific POR mutations, the presence of additional genetic or environmental factors, and management, include early death due to developmental malformations or adrenal crisis, bilateral oophorectomies after spontaneous rupture of ovarian macro-cysts, genital ambiguity, abnormal pubertal development, and nearly normal phenotype with successful pregnancy outcomes by assisted reproduction. Thus, timely diagnosis including prenatal diagnosis with invasive and non-invasive techniques and appropriate management is essential to improve patients’ outcomes. However, even in cases with conclusive diagnosis, comprehensive assessment is needed to avoid severe complications, such as chromosomal test to help sex assignment and evaluation of adrenal function to detect partial adrenal insufficiency. In recent years, it has been noted that proper hormone replacement therapy can lead to decrease or resolve of ovarian macro-cysts, and healthy babies can be delivered by in vitro fertilization and frozen embryo transfer following adequate control of multiple hormonal imbalances. Treatment may be complicated with adverse effects on drug metabolism caused by POR mutations. Unique challenges occur in female PORD patients such as ovarian macro-cysts prone to spontaneous rupture, masculinized genitalia without progression after birth, more frequently affected pubertal development, and impaired fertility. Thus, this review focuses only on 46, XX PORD patients to summarize the potential molecular pathogenesis, differential diagnosis of classic and non-classic PORD, and tailoring therapy to maintain health, avoid severe complications, and promote fertility.

## 1 Introduction

Cytochrome P450 oxidoreductase deficiency (PORD) is a rare type of congenital adrenal hyperplasia (CAH) inherited in an autosomal recessive manner caused by cytochrome P450 oxidoreductase (POR) mutations, which results in multiple hormonal imbalances with phenotypic diversity (1–5). For the protein encoded by POR gene located on chromosome 7 is a bi-flavoprotein and responsible for transferring electron from nicotinamide adenine dinucleotide phosphate (NADPH) to all 50 microsomal cytochrome P450 in the endoplasmic reticulum, including steroidogenic enzymes such as cytochrome P450 21A2 (CYP21A2), cytochrome P450 17A1 (CYP17A1), and aromatase (CYP19A1), and drug-metabolizing enzymes like cytochrome P450 3A4 (CYP3A4), cytochrome P450 2C9 (CYP2C9), and cytochrome P450 2C19 (CYP2C19), and some non-P450 enzymes; POR mutations can disturb steroidogenesis leading to adrenal and gonadal dysfunction and affect drug metabolism regulated by hepatic cytochrome p450 enzymes (3, 6–10). Potential insufficiency of cortisol increases episodes of illness and may lead to life-threatening adrenal crisis during stress (11). PORD is a group of disease with broad heterogeneity ranging from asymptomatic to skeletal malformations similar to Antley–Bixler syndrome (ABS), disorders of sex development (DSD), CAH phenotypes, and reduced reproductive capacity due to abnormal hormonal patterns (1, 12). Sporadic and familial cases with affected siblings have been reported (4, 13, 14). However, the exact prevalence of PORD is still unknown (13, 15).

The first presumed case of PORD was described as pseudohermaphroditism with multiple defects in steroid biosynthesis indicative of combined CYP21A2 and CYP17A1 deficiency in 1985 (1). However, the molecular etiology of POR mutations was not discovered until almost two decades later, which was compatible with the biochemical findings (4). Now, more than 100 patients with approximately 200 mutations and polymorphisms in POR have been reported (16–18). PORD can be divided into two forms based on severity and predominant symptoms: classic PORD with ABS and/or DSD phenotype, which is severe, and non-classic PORD, also termed as late- or adult-onset PORD, solely with menstrual disorders, infertility, and ovarian macro-cysts as primary manifestations, which are mild (4, 13, 19). The former can be subdivided into two types: PORD with ABS-like phenotype, which has skeletal malformations and is more severe even causing early death; and PORD with DSD and absence of ABS-like phenotype, which is less severe (20, 21). PORD with typical skeletal malformations is once categorized as phenotype II ABS, but now it is thought to be a separate entity distinguished from ABS (22–24).

Although with the development of molecular genetics and increasing understanding of the condition, many cases can be detected <3 months, several diagnostic challenges (especially for non-classic PORD) and management conundrum continue to exist owing to clinical variabilities and complex disease-related comorbidities and adverse effects related to treatment (2, 25). For example, airway obstruction due to choanal atresia and adrenal crisis resulting from insufficient production and action of cortisol may lead to life-threatening conditions. Yet, inappropriate sex assignment as a result of untimely diagnosis and bilateral oophorectomy during adolescents following rupture of ovarian cysts because of unsatisfactory control in hormonal imbalances were reported (4, 26). In addition, female patients with PORD present DSD and delayed pubertal development more frequently than affected male patients, and the influence of medical treatment on pregnancy needs to be accounted for (26). Ovarian adrenal rest tumors (OARTs) are less detectable than testicular adrenal rest tumors (TARTs) in CAH by conventional imaging (27). Thereby, diagnosis and management on PORD patients with female reproductive organs present unique challenges. Thus, this review focuses on 46,XX female patients, albeit PORD can occur and cause ambiguous genitalia in both sexes (28, 29).

## 2 Pathophysiology

**Figure 1:**
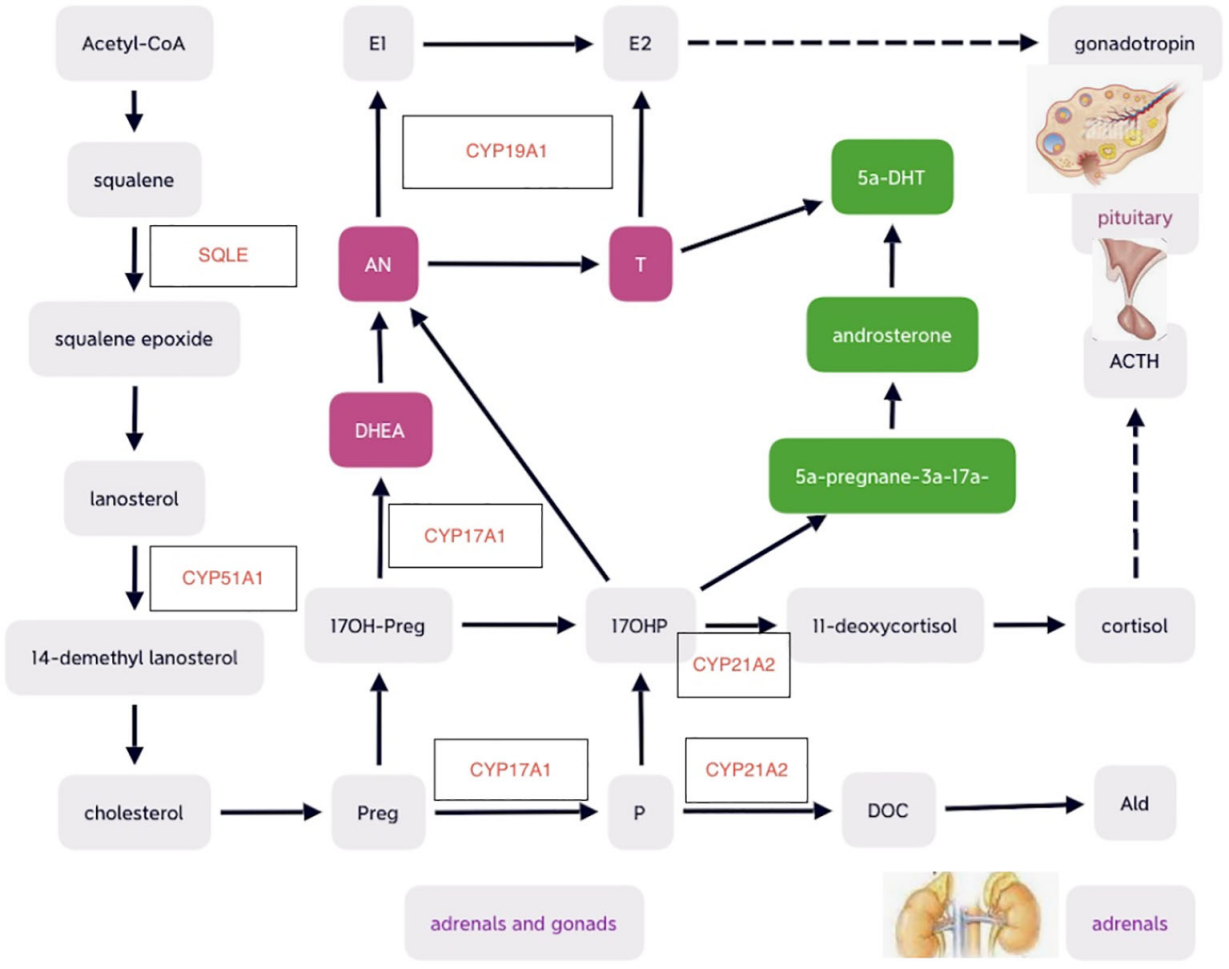
Schematic diagrams of cholesterol and steroid biosynthesis , including the canonical front pathway (in purple) and alternative ‘backdoor’ pathway (in green) for androgen production. Preg, pregnenolone; 17OH-Preg, 17 hydroxy-pregnenelone; DHEA, dihydroepiandrosterone; DHT, dihydrotestosterone; AN, androstenedione; T, testosterone; E2, estradiol; E1, estrone; P, progesterone; DOC, deoxycorticosterone; Ald, aldosterone; ACTH, adrenocorticotropic hormone. .

Many cytochrome P450 enzymes and some non-P450 enzymes require electron donation from POR for catalytic activities, including adrenal and gonadal CYP21A2, CYP17A1, CYP19A1, and lanosterol 14-alpha-demethylase (CYP51A1), and squalene epoxide (SQLE) involved in cholesterol and steroid metabolism (9, 30). Therefore, POR plays significant roles as essential redox partner in physiological homeostasis and hepatic drug metabolism (31, 32). POR mutations result in multiple imbalanced adrenal and gonadal hormones, which in turn lead to altered feedback on hypothalamus–pituitary axis (HPA) and hypothalamus–pituitary–gonadal (HPG) axis. Mutant POR decreased enzymatic activities of CYP17A1 and CYP19A1 and may cause low levels of estradiol (E2), which result in elevations of pituitary gonadotropins including follicle-stimulating hormone (FSH) and luteinizing hormone (LH). Defects of CYP21A2 secondary to POR mutations may lead to potential deficiency of cortisol and mild increase in pituitary production of adrenocorticotrophic hormone (ACTH) commonly without obvious signs of adrenal hyperplasia detected on adrenal imaging (16, 19). Although circulating testosterone (T) levels are usually low-normal during childhood, androgen excess may exist during fetal period, which may begin at early pregnancy and peak at mid-gestation and affect genital development (4). The predominant excess dihydrotestosterone (DHT), a potent androgen, during fetal period is speculated to convert from the elevated 17 hydroxy-progesterone (17OHP) with androsterone as the precursor of DHT through the alternative backdoor pathway bypassing the canonical pathway in which T is the precursor of dihydrotestosterone (DHT) (4) (as shown in **Figure 1**).

**Figure 2:**
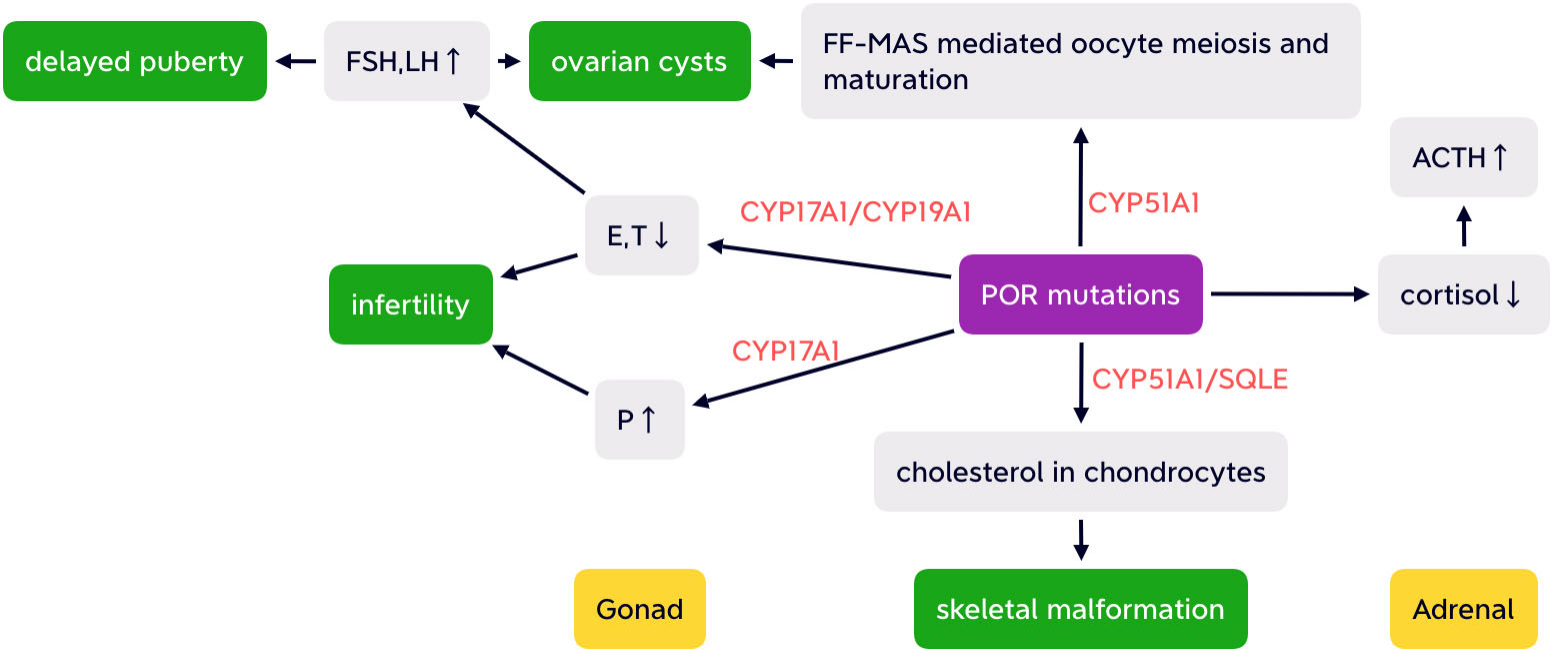
Simplified possible pathogenesis of ABS-like phenotype, partial adrenal insufficiency, delayed puberty, infertility, and ovarian cysts with altered HPA and HPO axis caused by POR mutations. FSH, follicular stimulating hormone; LH, luteinizing hormone; E, estrogen; T, testosterone; P, progesterone; FF-MAS, follicular fluid meiosis-activating sterols; CYP17A1,cytochrome P450 17A1; CYP19A1, aromatase; CYP51A1, cytochrome P450 51A1; SQLE, squalene epoxide; ACTH, adrenocorticotropic hormone.

Another electron-accepting enzyme CYP51A1 is highly expressed in human gonads and is essential for conversion of lanosterol (33). Follicular fluid meiosis-activating sterols (FF-MAS) produced from lanosterol participate in resumption of oocyte meiosis and maturation (34, 35). Thus, decreased CYP51A1 caused by POR mutation may lead to oocyte arrest, which may be associated with ovarian cyst development (36). The “double hit” of both elevated gonadotropins and reduced FF-MAS mediated oocyte maturation contribute to ovarian cysts (26) (as shown in **Figure 2**).

## 3 Pathogenesis

The decreased enzymatic activities of CYP21A2, CYP17A1, and CYP19A1 caused by POR mutations may lead to elevated 17OHP, progesterone (P), low estradiol (E2), and T, normal or elevated mineral corticoid such as deoxycorticosterone (DOC) level and normal or low levels of cortisol, which may show inadequate responsiveness to ACTH stimulation suggestive of partial adrenal insufficiency (13, 37). Potential cortisol deficiency may result in adrenal crises on stress (11).

### 3.1 Hypothesis for DSD and maternal virilization during pregnancy

Contradictive antenatal virilization and postnatal androgen deficiency and maternal virilization during pregnancy resolving rapidly after delivery can be observed in PORD (38). There are two hypothesis regarding the demonstrated specific fetal excess androgen: the selective backdoor pathway of androgen production in fetus and inhibition of placental CYP19A1 activity by mutant POR (39, 40). For the former, the accumulation of 17OHP by the fetal adrenals can activate the backdoor pathway leading to an overproduction of DHT without intermediacy of T, which is highly bioactive and can pass through the placenta to the maternal, possibly accompanied by other excess androgen metabolites (41). The alternative backdoor pathway closed soon after birth, so newborn virilization did not progress after birth, and maternal masculinization diminished rapidly after delivery. For the latter, excess fetal adrenal androgens, such as androstenedione and T derived from maternal dehydroepiandrosterone (DHEA), were converted to 5a-DHT instead of being aromatase to estrone (E1) and estriol (E3) in the placenta because of placental aromatase deficiency affected by POR mutations (4, 11, 12). Which attributes most to androgen excess during pregnancy remains debatable, with the latter as the major cause supported by Fluck et al. (39, 42).

### 3.2 Possible pathogenesis of ABS-like phenotype

Skeletal malformations in PORD may result from the impairment of enzymatic activity involved in cholesterol synthesis in chondrocytes catalyzed by CYP51A1 and SQMG requiring electrons from POR. Defects of both CYP51A1 and SQMG catalytic activities disturbed chondrocytic cholesterol production and consequently resulted in insufficient cell differentiation and increased apoptosis, which was supported by mouse knockout of *cyp51* resembling ABS (12, 43, 44) (as shown in **Figure 2**). Impaired activity of cytochrome P450 26B1 (CYP26B1), the retinoid acid (RA) grading enzyme, may also be associated with the ABS phenotype with excess RA leading to premature fusion of the cranial sutures and aberrant osteoblast–osteocyte transitioning (45–47).

### 3.3 Pathogenesis of delayed pubertal development and infertility

Decreased E2 and T may have a negative effect on pubertal development. No spontaneous pregnancy has been reported in PORD patients up to now (13, 48). POR mutations attenuate both the steroid- and drug-metabolizing enzymatic activities (39). Thereby, infertility may be attributed to imbalanced steroidogenesis, impaired endometrial receptivity, attenuation of drug metabolism, and the high risk of miscarriage (19, 48, 49). Anovulation due to defective follicular maturation with relatively low E2/T levels and atrophic endometrium resulting from the direct anti-proliferative effect of abnormally elevated P may be the main contributing factors of impaired reproductive capacities, whereas fertility potential of oocytes is still reserved (13, 48, 50).

Maybe genetic variability is also involved in the pathogenesis of PORD.

## 4 Clinical features

The activities of three steroidogenic enzymes may be affected to various degrees by POR mutations, which results in broad spectrum of manifestation, from asymptomatic to combined with ABS and DSD phenotype (48). The predominant symptomatology may vary corresponding to the severity of the complex set of hormonal derangements and specific stages of life, with some presenting prenatally and some phenotypically normal until adult. The predominant anomalies were listed as follows according to the developmental stages and clinical findings.

1. In uterus, fetal skeletal malformations may be visualized on ultrasonography, including ABS-like discoveries of craniocynostosis, protruding forehead, choanal atresia, radio-humeral synostosis, and femoral shortening and bowing, club feet, with femoral changes readily revealed (12, 51). To systematically assess the severities of skeletal malformations, Krone et al. developed a clinical scoring system consisting of six domains: craniosynostosis (3’), midface hypoplasia (3’), hand and feet malformations (3’), large joint synostosis (3’), femoral bowing (2’), and another additional malformations (2’) with total maximum score (TMS) from 0 to 16 (52). During pregnancy the other symptom is maternal virilization, which usually manifests from mid-gestation (signs of acne, hirsutism, voice deepening, and acromegaly) and decreased a few weeks after delivery (29). In a case report of PORD, maternal acne and voice deepening was noted at 14–16w and 24w, respectively (29). Alertness of these features is helpful for the identification of possible PORD.

2. After birth during infancy or childhood, signs of DSD, the most common symptom of PORD including ambiguous genitalia even with Prader V, may be revealed in classic PORD, although a timely conclusive diagnosis may not be established (2, 26, 53). Labial fusion, clitoromegaly, vaginal atresia, and a single urogenital orifice had been described in PORD (54). Recurrent otitis media and conductive hearing loss were described in PORD, which may result from stenotic external auditory canals and middle or inner ear malformations (15). In recent years, with the awareness of the condition, some distinctive subtle facial abnormalities were also documented, including lower eyelid fat pads, prominent lower eyelid-zygoma transverse line, single earlobe crease, and underdeveloped or absent anti helix (12).

3. During adolescent, absent or delayed puberty, menstrual disorders, and infertility may be demonstrated, which can be the primary manifestations of PORD or occur with ABS or DSD simultaneously (13, 28, 55). It can manifest with primary or secondary amenorrhea, oligomenorrhea, irregular menses, disordered pubertal development, and primary infertility. Delayed puberty may be correlated with subclinical hypoglycemia due to slight adrenal insufficiency as well as insufficient sex hormones (2).

Another common finding in PORD is ovarian macro-cysts, especially for the non-classic form, which is prone to spontaneous rupture leading to emergent surgery and loss of reproductive capacity (56). The possible associated symptoms may range from mild abdominal discomfort to acute abdominal pain (13, 37). Serial bilateral ovariectomies owing to rupture of ovarian cysts had been noted in adolescent patients (4, 26). Of note, large ovarian cysts can even be detected during infancy (28, 57).

Besides the typical presentations of ABS, DSD, maternal virilization during pregnancy in classic PORD, menstrual disorders, and ovarian cysts in non-classic PORD and infertility in both forms, less common manifestations were also demonstrated, such as adrenal crisis and hypertension. Although most PORD cases present with partial adrenal insufficiency, adrenal crisis occurred in a 46, XX patient with Y578C/I444fsX449 mutations (56). In addition arterial hypertension of 130/54mmHg in a 5-year-old child and 140-160/90-95mmHg in an adult were reported (15, 25). The mildly hypertensive condition is possibly secondary to elevated DOC (52).

To sum up, the clinical findings include ABS-like skeletal malformations, maternal virilization during pregnancy, DSD, absent or delayed puberty, menstrual disorders, ovarian macro-cysts, infertility, adrenal insufficiency, hypertension, recurrent otitis media, and conductive hearing loss.

## 5 Biochemical assessments

As 21-hydroxylase deficiency (21-OHD) only affects adrenal steroidogenesis and 17 alpha-hydroxylase involves in both adrenal and gonadal steroid production and POR mutations have disruptive effects on both CYP21A2 and CYP17A1, multiple hormonal derangements can be observed in PORD by serum and urinary sampling analysis, reflecting altered steroid biosynthesis and metabolism (58). Several other tests are needed to establish definitive diagnosis and assist management.

### 5.1 Chromosomal test

As genital ambiguity may occur in both sexes in PORD, chromosomal test is essential for gender assignment (59). It has been reported that some 46, XX PORD patients with ambiguous external genitalia were initially raised as boys (26).

### 5.2 Evaluation of steroid hormone profiling

The serum 17OHP, P, and 17 hydropregnenolone levels are usually elevated, and basal cortisol and ACTH levels may be normal or nearly normal (26, 56). Serum E2 concentrations may be normal or low with normal or elevated FSH and LH levels (54). Serum T levels are usually low-normal, different from that in ABS and polycystic ovarian syndrome (PCOS) (13).

### 5.3 ACTH stimulation test

Although patients with PORD usually have normal levels of basal cortisol, they are still at a relatively high risk of adrenal insufficiency primarily due to deficient action of glucocorticoid, especially during stress (60). An overlook of adrenal insufficiency may lead to disastrous consequences including untimely death (11, 26). It had been revealed that unexplained shock appeared in a 8-year-old highly suspected PORD patient after routine appendectomy (26). ACTH stimulation test is recommended in PORD patients, as basal cortisol is not sensitive to assess potential adrenal insufficiency, especially during inflammatory stress, peri-operation, and episodes of illnesses (23). A marked increase in 17OHP and P and inadequate increase in cortisol can be observed after cosyntropin test (13, 37). In a Japanese study of PORD, the basal cortisol level was <8μg/dl and <18μg/dl after ACTH stimulation, demonstrating partial adrenal insufficiency (2).

### 5.4 Ovarian stimulation test

PORD patients also showed a special pattern to ovarian stimulation. Compared with common *in vitro* fertilization (IVF) cycles, in PORD, it presents with modestly increase in serum E2 and an unusual elevation of P following letrozole combined with human menopausal gonadotropin (hMG) protocol or gonadotropin-releasing hormone (GnRH) agonist and GnRH antagonist regimen (13, 61).

### 5.5 Urine steroid metabolite analysis

Urine steroid profile may reveal partially combined deficient CYP21A2 and CYP17A1 activities resembling that in serum (21). The ratios of steroid substrate metabolite to product metabolite can allow diagnosis of a variety of steroidogenic disorders with the elevated ratio of P metabolite pregnanediol over cortisol metabolites (tetrahydrocortisone (THE)+tetrahydrocortisol (THF)+5aTHF) as a PORD-specific diagnostic method (52). Urine androsterone derived from both front door and backdoor pathway and etiocholanolone derived almost exclusively from front door pathway during early infancy (62).

### 5.6 Genetic analysis

In patients combined with manifestations of 21OHD and 17OHD, corresponding gene tests including CYP21A2, CYP17A1, CYP19A1, and POR are beneficial for establishing molecular diagnosis.

## 6 Genotype–phenotype correlations

Correlations between POR mutations and the clinical and biochemical phenotype contribute to elucidate the molecular basis of PORD pathogenesis, provide valuable information for genetic counseling, and predict clinical manifestations and prognosis (24, 52). Genotype–phenotype correlation is a hot spot in many genetic disorders and the same is true in PORD. It remains a conundrum, albeit great developments emerged on how mutant POR had deleterious effects on the microsomal enzymes and interactions between different redox partners *via* computational analysis of available data and functional studies *in vitro*. POR mutations cause variable effects on the enzymatic activities with different redox partners (24). For example, the unique founder mutations of different ethnic group A287P (Caucasian), R457H (Japanese), and P399_E401 (Turkish) showed various degrees of impaired enzyme activity with retaining residual 21-hydroxylase activity, 70%, 32%, –; 17 hydroxylase activity, 20%, 3%, and 24%; 17, 20 lyase potent 10%, –, 31%; and CYP19A1, 60%, 1%, and 25% (21, 31, 32, 39, 55, 63). Distinct mutations in different domains had also been described, such as Y181D affecting flavin mononucleotide (FMN) binding, R457H, Y459H, V492E in flavin adenine dinucleotide (FAD) binding, and C569Y, V608F in NADPH binding domain (18, 21, 55, 62–66). Generally, the clinical manifestations of PORD correlated well with the degree of impairment of POR enzymatic activity (12). Patients with null or splicing mutations present more severely than those with missense mutations (52, 54). POR expression as early as a two-cell stage indicates its important role in cellular function (67). Hence, patients with null mutations on both POR alleles have not been described yet, probably due to its incompatibility to life, which is consistent with observation of embryonic lethality in murine *por* knockout model (67, 68). However, it has been discovered in recent years that even patients harboring identical POR mutations present with impaired enzymatic activity to a distinct degree. In two affected siblings with compound heterozygous mutations (p.Gln609*/p.Trp620Ser), the older sister presented with primary amenorrhea without signs of DSD whereas the younger manifested with mild genital virilization and labial fusion and irregular menses (13). The discordant presentations of the same mutation demonstrated that phenotypic expression in PORD was multifactorial and may be influenced by other genetic, epigenetic, or environmental factors, such as the regulation of *Drosophila* mothers against decapentaplegic protein (Smad3/4), thyroid receptors, and transcription factor activating protein 2 (AP-2) in human POR transcription (3, 69). Susceptibility to xenobiotics may change either resulting from impaired hepatic enzymes secondary to POR mutations (25). Furthermore, it is proposed that POR polymorphisms, cytochrome P450, and associated genes may also involve in the diverse PORD phenotype (39).

## 7 Diagnosis and differential diagnosis

The typical cases can be diagnosed during infancy or childhood at pediatrics, usually <3 months, while non-classic PORD patients can be referred >30 years of old (2, 13). By carefully obtaining medical history, physical examination, and the choice of biochemical steroid profiling and imaging tests, it is not difficult to identify PORD cases with manifest clinical and biochemical characteristics, such as maternal virilization and low levels of E3 during pregnancy, ABS-like phenotype, signs of DSD, menstrual disorders, primary infertility, recurrent ovarian macrocysts prone to rupture, and combined steroidogenic enzymatic deficiency. Some cases can be detected in neonatal screening program (56, 57). However, diagnostic challenges still exist because of overlapping phenotypes with other disorders, paucity and unawareness of the condition, and untypical and non-specific presentations in mild cases (13). It needs to be differentiated with the following entities: other forms of CAH, such as 21-OHD, CYP17A1, and CYP19A1 deficiency, PCOS, and primary ovarian insufficiency (POI) (12, 15, 16).

### 7.1 ABS

Both PORD and ABS can present with similar malformations such as craniosynostosis, midface hypoplasia, radiohumeral synostosis, and femoral bowing, but ABS is an autosomal dominant inherited disease caused by fibroblast growth factor receptor (FGFR) mutations with normal steroid production (55, 70, 71). ABS combined with abnormal steroidogenesis is now categorized as PORD (18). Steroid profiling and gene testing can help distinguish the two entities.

### 7.2 CAH

Some PORD cases with abnormal external genitalia and increased 17OHP and P were reported to be initially regarded as 21-OHD or CYP19A1 deficiency. Differentiation of the distinct forms of CAH is important for physiological dose of glucocorticoid is needed without progression of excess androgen excretion after birth in PORD, whereas a supra-physiological cortisol replacement is required to suppress androgen excess in 21-OHD. Clinically, virilization in PORD did not progress postnatally, opposite to that of untreated 21-OHD (5, 28, 72). Biochemically the amplitude of elevation of 17OHP was lower than that of P in PORD cases, which is opposite in 21-OHD (52). Besides the impairment of CYP21A2 in both conditions, CYP17A1 activity is also impacted by POR mutations, resulting in low-normal levels of E2/T, in contrast with 21-OHD. Cortisol levels may be normal but responds poorly to ACTH stimulation in PORD, which is commonly low in 21-OHD. Aldosterone deficiency with salt-wasting is only detected in 21–OHD, not in PORD, albeit theoretically possible (37). Biochemical measurement of urinary steroid metabolites can differentiate PORD from 21-OHD, including the pregnanetriolone/tetrahydrocortisone ratio (Ptl/THEs), tetrahydroaldosterone (THAldo), pregnenediol (PD5), 11OHAn/THAldo or 11OHAn/PD5 ratio, and 11β-hydroxyandrosterone (11OHAn), which is confirmed in Japanese infants (73). OART has been described in cases with 21 OHD, whereas to date, no OART was reported in PORD (27).

### 7.3 PCOS

Non-classic or adult-onset PORD should also be differentiated from PCOS, as they can both present with irregular menses, infertility, and ovarian cysts (16). However, in PORD, serum T is low to normal afterbirth with elevated P concentrations, and the ovarian cysts are usually at the risk of spontaneous rupture requiring surgery, while in PCOS, hyperandrogenism often exists with normal P levels and polycystic ovarian morphological features of a number of antral follicles (2–9 mm in diameter), which seldomly require operation (28, 74–76).

### 7.4 POI

Secondary amenorrhea with decreased E2 and anti-Müllerian hormone (AMH) and in turn increased FSH and LH, which were shown in some non-classic PORD, can lead to suspicion of POI. Steroid hormone and genetic assays are helpful for the differentiation (13).

Because of the broad heterogenous phenotypic spectrum of PORD, maybe some asymptomatic or mild cases remain undiagnosed with some misdiagnosed because of ascertainment bias (13, 28, 77). POR gene testing may help establish diagnosis if no mutations were found in genes that encode proteins directly catalyzing steroidogenesis in cases with disordered steroid hormones (78). Comprehensive evaluation is essential after diagnosing PORD to provide proper management, such as assessment of chromosomal karyotype and adrenal insufficiency, which may lead to life-threatening adrenal crisis if overlooked (26) (as shown in **Table 1**).

**Table 1:** The essential comprehensive evaluation in suspected PORD cases and the relevant key points in management.

Essential comprehensive evaluation	Key points in management of PORD
Chromosomal test	Sex assignment
ACTH stimulation test	Glucocorticoid supplement therapy
ABS-like phenotype	Multiple interdisciplinary operations
DSD	Early correction, usually <2 years old
Pelvic imaging for ovarian cysts	E2 replacement, sometimes plus GnRH analog and corticosteroids
Pubertal development	Sex hormone replacement therapy
Infertility	IVF-FET
Education	Psychosocial support

ACTH, adrenocorticotropic hormone; ABS, Antley–Bixler syndrome; DSD, disorders of sex development; E2, estradiol; GnRH, gonadotropin releasing hormone; IVF-FET, in vitro fertilization and frozen embryo transfer.

## 8 Prenatal screening and diagnosis

Prenatal screening and subsequent diagnosis of suspected PORD fetus is essential for optimum management of the condition. The routine antenatal examinations can provide useful clues for identification of PORD, such as low maternal E3 levels and skeletal malformations including choanal atresia and femoral bowing detected on ultrasonography (28, 51). Maternal serum E3 was proposed as a marker steroid for PORD (79). Maternal virilization during pregnancy may occur with 46, XX PORD cases (80). Of note, maternal masculinization can present with excess androgens caused by other causes (81). Low E3 occurs on the presence of several other steroidogenic deficiency, such as steroidogenic acute regulatory protein deficiency, CYP17A1 deficiency, steroid sulfates deficiency, defects of placental aromatase, hypopituitarism, and Smith–Lemli–Opitz syndrome. ABS-like phenotype can be observed in multiple syndromes, including trisomy 18 syndrome, Apert, Jacobse, Jackson–Weiss, and Pfeiffer syndrome (51). Although among maternal virilization, low serum E3, and ABS-like phenotype, none of the clinical, laboratory, and sonographic findings are PORD specific, we propose that two of three otherwise unexplained features are highly indicative of PORD, which needs further investigation in clinical practice. Importantly, perinatologists should be aware of and remain alert to the positive findings, which significantly implicates pregnancy outcome and fetal prognosis. Maternal urinary steroid profiling is also helpful for the prenatal diagnosis with excessive excretion of epiallopregnanediol of largely fetal origin in maternal urine (82).

Prenatal diagnosis may be needed in highly suspicious cases of PORD. Amniocentesis could be done to detect the underlying genetic etiology during second trimester (51). Nowadays, a non-invasive technique has been developed to obtain fetal genetic information following the discovery of fetal cell-free DNA in maternal circulation, which allows the diagnosis of single gene disorders at an early gestational age (83, 84).

## 9 Treatment

For the paucity and newly identified traits of PORD, knowledge regarding optimum management is limited, and there is no clear guidelines for treatment globally (13, 16). Therefore, current therapies for PORD are commonly dependent upon clinicians’ experience and individualized. Maybe the primary goals are control of multiple hormonal imbalances, correction of developmental malformations, facilitation of pubertal development, and treatment and prevention of severe complications including adrenal crisis and rupture of ovarian macro-cysts, fertility facilitation, and psychosocial support, especially for sex assignment. Management challenges still emerged including intrauterine therapy, the effect of POR mutations on clinically used drugs, and life-threatening adrenal crisis, albeit some therapeutic advances have achieved, e.g., successful pregnancy outcomes following PORD and effective therapy for ovarian cyst without operation. Furthermore, treatment is complicated by disease-related comorbidities such as upper respiratory obstruction and treatment-related adverse effects (e.g., the risk of breast cancer) (25).

The following issues need to be considered in the treatment for PORD according to the corresponding comprehensive evaluation during and after diagnosis of PORD (as shown in **Table 1**):

### 9.1 Glucocorticoid replacement

According to a meta-analysis of PORD of both sexes, in the genetic-proven PORD patients, only 54% underwent assessment of adrenal insufficiency with a positive rate of 78% (38/49), implying a rather common phenomenon in PORD (17). Thus, glucocorticoid replacement is required in a majority of PORD patients with permanent cortisol replacement therapy or at least stress dose cover, which is somewhat contrast to what was suggested that hydrocortisone treatment may be avoided in some mild to moderate cases (2, 52). Glucocorticoid replacement may be essential in the following conditions: episodes of illnesses, inflammatory stress, peri-operative stage, unsatisfactory control of ovarian macro-cysts, and prior to frozen embryo transfer (ET) (11, 13, 28, 85). During delivery, the administration of intravenous injection of hydrocortisone (100 mg) was also demonstrated (13). In infants and children with PORD, short-acting glucocorticoid, such as hydrocortisone, is prescribed for its minimum effect on growth, whereas both short- and long-acting glucocorticoids can be selected in adults. For example, hydrocortisone (25–30 mg) or dexomethasone (0.375mg, 0.5mg, and 0.75mg) were used to suppress elevated P levels and aid in successful embryo implantation (48). Commonly replacement of physiological dose of glucocorticoid is required in PORD, different from that of 21-OHD, which needs supra-physiological dose of cortisol to suppress adrenal androgen excess. Glucocorticoid supplement can also lead to normalization of blood pressure, which was reported in a 5-year-old child treated with oral hydrocortisone (6.25mg/m^2^/d) (15). It is essential to adjust glucocorticoid doses during intercurrent illness, oxidase stress, and medical or dental procedures, as nearly 50% patients with adrenal insufficiency possibly suffer adrenal crisis partially due to the failure of replicating circadian rhythm of cortisol (86, 87).

### 9.2 Correction of developmental malformations

Skeletal abnormalities in PORD can be treated with staged orthopedic management (37, 88). Among these choanal obstruction (stenosis or atresia), one of the main causes of mortality in PORD requires timely surgical correction including nasal stints or tracheotomy and tracheostomy as needed (89). For craniosynostosis like in other craniofacial syndromes, multiple interdisciplinary reconstructive surgeries may be needed ranging from infancy to adolescence, as reported in a non-classic PORD patient serial surgeries were performed at 11 months and 8 years (13, 90). Operations for genital abnormalities were usually carried out before 2 years of age including feminizing genitoplasty such as cliteroplasty and vaginal reconstruction (11, 15, 91), although sometimes partial clitoral reduction and vaginoplasty were performed between 5 and 12 years of age because of opposite gender assignment (26). Anesthetic risks associated with ABS phenotype should be taken into account such as difficulties in intubation with choanal obstruction (92).

### 9.3 Sex assignment and psychosocial support

Proper sex assignment is pivotal in patients with DSD. In some cases, wrong gender identification was implemented, occasionally with subsequent administration of testosterone enanthate, which will have negative effect on children’s physical and psychosocial health (11). PORD exclusively affects steroidogenesis and does not alter gonadal morphogenesis, so PORD patients with 46, XX karyotype present with female reproductive organs including uterus and ovaries. Therefore, 46, XX PORD patient should be raised as girls despite the masculinized external genitalia. Maybe chromosomal test and pelvic imaging is helpful for correct sex assignment.

### 9.4 Developmental facilitation

Incomplete or absent pubertal development due to defects of sex steroid is common in PORD, which can be treated with E2 supplement (28). The initial ages with sex hormone replacement varied in different reports, with equine estrogens and P at 17, 13 years and 15, 11 years with E2 replacement therapy (11, 15, 25, 26). Of note, breast cancer without family history was elucidated in a 37-year-old PORD patient treated with sex hormone replacement for 20 years, possibly due to excess E2 exposure secondary to the impact of POR mutation on corresponding hepatic cytochrome p450 enzymes. Hence, drug metabolization should be accounted for in the administration of PORD, which also have impacts on hepatic drug-metabolizing enzymes, including CYP3A4 (93).

### 9.5 Treatment for ovarian cysts

Although ovarian cysts are benign tumors according to histological findings, the fact that they apt to rupture spontaneously and readily recur highlights the importance of timely and appropriate treatment (26). E2 replacement can markedly reduce the size of ovarian cysts, while E2/P, including combined oral contraceptives, sequential therapy combined with GnRH analog, and low-dose corticosteroids were needed in some patients (16, 22). It has been reported that administration of triptorelin (monthly intramuscular injection of 3.75mg for 2 months) on the basis of sex hormone replacement successfully reduced the number and size of ovarian cysts (13).

### 9.6 Treatment for infertility

No spontaneous pregnancy has been reported in PORD women, but successful pregnancy can be achieved by *in vitro* fertilization-frozen embryo transfer (IVF-FET) following adequate hormonal control. Artificial endometrial preparation was essential for low levels of E2 and elevated P altered endometrial receptivity, and administration of oral glucocorticoids. Twins live birth (a healthy boy and a healthy girl) were delivered at 37 + 2 weeks by cesarean section in a Chinese patient aged 29 with a compound heterozygous mutation of R457H/P399_E401del (48). Thus, the developmental potential of oocytes seemed not to be impaired by POR mutation. To date, only five cases with successful pregnancy outcomes (two twin live births and three singleton live births) in PORD, and of note, all of them had elevated 17OHP and basal P concentrations and ovarian cysts, but none of them had clinical or biochemical obvious signs of adrenal insufficiency (13, 19, 48, 61). Several early pregnancy losses after FET were also reported in a non-classic PORD patient (c.1631T>C/c.1723G>A), probably associated with dysfunctional steroidogenesis and disordered immune system (19).

### 9.7 Intrauterine intervention

Intrauterine treatment or termination of pregnancy (TOP). Management dilemma exists in cases prenatally diagnosed and encountered continue pregnancy and intrauterine treatment or TOP. In fetus with severe skeletal malformations, TOP may be a choice after definitive diagnosis and genetic counseling (51). Once virilization was observed during pregnancy in a 46,XX fetus, it is proposed to decline DHT promptly to prevent further development of abnormal genitalia.

## 10 Prognosis

The proper intervention of PORD may require participation of multidisciplinary practitioners, including sonologists, perinatologists, pediatricians, gynecologists, obstetricians, and endocrinologists. The prognosis is associated with early diagnosis, severity of bony malformations, timely perinatal care, compliance to treatment, and reinforced education (26, 29, 37, 94). Good clinical outcomes also call for education of both healthcare providers, patients, and their parents, especially in transition from pediatrics to adult clinics (94). Anyway, it has been reported that quality of life (QOL) of patients presenting with DSD may be not impaired after proper management (95).

## 11 Conclusion

In summary, PORD should be considered in patients with ABS phenotype, DSD, or menstrual disorders combined with steroidogenic abnormalities (96). Establishing the correct diagnosis early is essential for proper management, such as proper sex assignment and tailored glucocorticoid doses. The prevention of adrenal crisis should be kept in mind in PORD, albeit usually with normal basal cortisol levels, as adrenal insufficiency resulting from poor actions of cortisol can be life threatening during stress. The control of ovarian cysts is pivotal to reserve reproductive potent because of the traits of spontaneous rupture requiring oophorectomy. Infertility can be achieved by IVF-FET following adequate treatment of multiple imbalanced hormones. Prenatal screening and diagnosis allow timely intervention and prevent severe birth defects. Thus, prompt diagnosis and tailoring therapy based on thorough evaluation is beneficial to improve clinical outcomes in patients with PORD. Although molecular mechanisms in PORD have been comprehensively investigated with great advancement, there is still a long way to go to identify genotype–phenotype correlations in PORD complicated by possible involvement of other genes, epigenetic, and environmental factors, which may be explored in future research. Investigating potential therapeutic targets for PORD may also provide future direction for better management of the condition.

## Author contributions

All authors listed have made a substantial, direct, and intellectual contribution to the work and approved it for publication.

## References

[1] Peterson RE Imperato-McGinley J Gautier T Shackleton C. Male pseudohermaphroditism due to multiple defects in steroid-biosynthetic microsomal mixed-function oxidases. A new variant of congenital adrenal hyperplasia. N Engl J Med (1985) 313(19):1182–91. doi: 10.1056/NEJM198511073131903 2932643

[2] Yatsuga S Amano N Nakamura-Utsunomiya A Kobayashi H Takasawa K Nagasaki K. Clinical characteristics of cytochrome P450 oxidoreductase deficiency: a nationwide survey in Japan. Endocr J (2020) 67(8):853–7. doi: 10.1507/endocrj.EJ20-0011 32321882

[3] Miller WL. P450 oxidoreductase deficiency: a disorder of steroidogenesis with multiple clinical manifestations. Sci Signal (2012) 5(247):pt11. doi: 10.1126/scisignal.2003318 23092891

[4] Arlt W Walker EA Draper N Ivison HE Ride JP Hammer F. Congenital adrenal hyperplasia caused by mutant P450 oxidoreductase and human androgen synthesis: analytical study. Lancet (2004) 363(9427):2128–35. doi: 10.1016/S0140-6736(04)16503-3 15220035

[5] Krone N Dhir V Ivison HE Arlt W. Congenital adrenal hyperplasia and P450 oxidoreductase deficiency. Clin Endocrinol (Oxf) (2007) 66(2):162–72. doi: 10.1111/j.1365-2265.2006.02740.x 17223983

[6] Moutinho D Marohnic CC Panda SP Rueff J Masters BS Kranendonk M. Altered human CYP3A4 activity caused by Antley-Bixler syndrome-related variants of NADPH-cytochrome P450 oxidoreductase measured in a robust in *vitro* system. Drug Metab Dispos (2012) 40(4):754–60. doi: 10.1124/dmd.111.042820 PMC3310424 22252407

[7] Miller WL. Steroidogenic electron-transfer factors and their diseases. Ann Pediatr Endocrinol Metab (2021) 26(3):138–48. doi: 10.6065/apem.2142154.077 PMC8505039 34610701

[8] Velazquez MNR Parween S Udhane SS Pandey AV. Variability in human drug metabolizing cytochrome P450 CYP2C9, CYP2C19 and CYP3A5 activities caused by genetic variations in cytochrome P450 oxidoreductase. Biochem Biophys Res Commun (2019) 515(1):133–8. doi: 10.1016/j.bbrc.2019.05.127 31128914

[9] Aguilar A Wu S De Luca F. P450 oxidoreductase expressed in rat chondrocytes modulates chondrogenesis *via* cholesterol- and Indian Hedgehog-dependent mechanisms. Endocrinology (2009) 150(6):2732–9. doi: 10.1210/en.2009-0043 19264869

[10] Pandey AV Flück CE Huang N Tajima T Fujieda K Miller WL. P450 oxidoreductase deficiency: a new disorder of steroidogenesis affecting all microsomal P450 enzymes. Endocr Res (2004) 30(4):881–8. doi: 10.1081/erc-200044134 15666840

[11] Fukami M Horikawa R Nagai T Tanaka T Naiki Y Sato N. Cytochrome P450 oxidoreductase gene mutations and Antley-Bixler syndrome with abnormal genitalia and/or impaired steroidogenesis: molecular and clinical studies in 10 patients. J Clin Endocrinol Metab (2005) 90(1):414–26. doi: 10.1210/jc.2004-0810 15483095

[12] Adachi M Tachibana K Asakura Y Yamamoto T Hanaki K Oka A. Compound heterozygous mutations of cytochrome P450 oxidoreductase gene (POR) in two patients with Antley-Bixler syndrome. Am J Med Genet A (2004) 128A(4):333–9. doi: 10.1002/ajmg.a.30169 15264278

[13] Papadakis GE Dumont A Bouligand J Chasseloup F Raggi A Catteau-Jonard S. Non-classic cytochrome P450 oxidoreductase deficiency strongly linked with menstrual cycle disorders and female infertility as primary manifestations. Hum Reprod (2020) 35(4):939–49. doi: 10.1093/humrep/deaa020 32242900

[14] Li H Dong R Zhang K Lyu Y Gao M Gai Z. Clinical and genetic analysis of a pedigree affected with cytochrome P450 oxidoreductase deficiency. Zhonghua Yi Xue Yi Chuan Xue Za Zhi (2020) 37(9):1005–8. doi: 10.3760/cma.j.cn511374-20190912-00468 32820517

[15] Lee Y Choi JH Oh A Kim GH Park SH Moon JE. Clinical, endocrinological, and molecular features of four Korean cases of cytochrome P450 oxidoreductase deficiency. Ann Pediatr Endocrinol Metab (2020) 25(2):97–103. doi: 10.6065/apem.1938152.076 32615689 PMC7336261

[16] Bai Y Li J Wang X. Cytochrome P450 oxidoreductase deficiency caused by R457H mutation in POR gene in Chinese: case report and literature review. J Ovarian Res (2017) 10(1):16. doi: 10.1186/s13048-017-0312-9 28288674 PMC5348910

[17] Dean B Chrisp GL Quartararo M Maguire AM Hameed S King BR. P450 oxidoreductase deficiency: A systematic review and meta-analysis of genotypes, phenotypes, and their relationships. J Clin Endocrinol Metab (2020) 105(3):dgz255. doi: 10.1210/clinem/dgz255 31825489

[18] Pandey AV Flück CE. NADPH P450 oxidoreductase: structure, function, and pathology of diseases. Pharmacol Ther (2013) 138(2):229–54. doi: 10.1016/j.pharmthera.2013.01.010 23353702

[19] Li Y Zhang CL Zhang SD. Infertility treatment for Chinese women with P450 oxidoreductase deficiency: Prospect on clinical management from IVF to FET. Front Endocrinol (Lausanne) (2022) 13:1019696. doi: 10.3389/fendo.2022.1019696 36619579 PMC9813486

[20] Bonamichi BD Santiago SL Bertola DR Kim CA Alonso N Mendonca BB. Long-term follow-up of a female with congenital adrenal hyperplasia due to P450-oxidoreductase deficiency. Arch Endocrinol Metab (2016) 60(5):500–4. doi: 10.1590/2359-3997000000213 PMC10118638 27737328

[21] Flück CE Tajima T Pandey AV Arlt W Okuhara K Verge CF. Mutant P450 oxidoreductase causes disordered steroidogenesis with and without Antley-Bixler syndrome. Nat Genet (2004) 36(3):228–30. doi: 10.1038/ng1300 14758361

[22] McCall AA Kirsch CF Ishiyama G Ishiyama A. Otologic findings in Antley-Bixler syndrome: a clinical and radiologic case report. Int J Pediatr Otorhinolaryngol (2007) 71(7):1139–43. doi: 10.1016/j.ijporl.2007.02.016 17482285

[23] Reardon W Smith A Honour JW Hindmarsh P Das D Rumsby G. Evidence for digenic inheritance in some cases of Antley-Bixler syndrome? J Med Genet (2000) 37(1):26–32. doi: 10.1136/jmg.37.1.26 10633130 PMC1734444

[24] Parween S Roucher-Boulez F Flück CE Lienhardt-Roussie A Mallet D Morel Y. P450 oxidoreductase deficiency: loss of activity caused by protein instability from a novel L374H mutation. J Clin Endocrinol Metab (2016) 101(12):4789–98. doi: 10.1210/jc.2016-1928 27603900

[25] ToMalik-Scharte D Maiter D Kirchheiner J Ivison HE Fuhr U Arlt W. Impaired hepatic drug and steroid metabolism in congenital adrenal hyperplasia due to P450 oxidoreductase deficiency. Eur J Endocrinol (2010) 163(6):919–24. doi: 10.1530/EJE-10-0764 PMC2977993 20844025

[26] Idkowiak J O'Riordan S Reisch N Malunowicz EM Collins F Kerstens MN. Pubertal presentation in seven patients with congenital adrenal hyperplasia due to P450 oxidoreductase deficiency. J Clin Endocrinol Metab (2011) 96(3):E453–62. doi: 10.1210/jc.2010-1607 PMC3124345 21190981

[27] Yildiz M Bayram A Bas F Karaman V Toksoy G Poyrazoglu S. Ovarian and paraovarian adrenal rest tumors are not uncommon in gonadectomy materials of historical congenital adrenal hyperplasia cases in childhood. Eur J Endocrinol (2022) 187(1):K13–8. doi: 10.1530/EJE-21-0913 35550562

[28] Sahakitrungruang T Huang N Tee MK Agrawal V Russell WE Crock P. Clinical, genetic, and enzymatic characterization of P450 oxidoreductase deficiency in four patients. J Clin Endocrinol Metab (2009) 94(12):4992–5000. doi: 10.1210/jc.2009-1460 19837910 PMC2795645

[29] Reisch N Idkowiak J Hughes BA Ivison HE Abdul-Rahman OA Hendon LG. Prenatal diagnosis of congenital adrenal hyperplasia caused by P450 oxidoreductase deficiency. J Clin Endocrinol Metab (2013) 98(3):E528–36. doi: 10.1210/jc.2012-3449 PMC3708032 23365120

[30] Panda SP Guntur AR Polusani SR Fajardo RJ Gakunga PT ROman LJ. Conditional deletion of cytochrome p450 reductase in osteoprogenitor cells affects long bone and skull development in mice recapitulating antley-bixler syndrome: role of a redox enzyme in development. PloS One (2013) 8(9):e75638. doi: 10.1371/journal.pone.0075638 24086598 PMC3783497

[31] Flück CE Mallet D Hofer G Samara-Boustani D Leger J Polak M. Deletion of P399_E401 in NADPH cytochrome P450 oxidoreductase results in partial mixed oxidase deficiency. Biochem Biophys Res Commun (2011) 412(4):572–7. doi: 10.1016/j.bbrc.2011.08.001 21843508

[32] Burkhard FZ Parween S Udhane SS Flück CE Pandey AV. P450 Oxidoreductase deficiency: Analysis of mutations and polymorphisms. J Steroid Biochem Mol Biol (2017) 165(Pt A):38–50. doi: 10.1016/j.jsbmb.2016.04.003 27068427

[33] Rozman D. Lanosterol 14alpha-demethylase (CYP51)–a cholesterol biosynthetic enzyme involved in production of meiosis activating sterols in oocytes and testis–a minireview. Pflugers Arch (2000) 439(3 Suppl):R56–7. doi: 10.1007/s004240000090 10653142

[34] Jin S Zhang M Lei L Wang C Fu M Ning G. Meiosis activating sterol (MAS) regulate FSH-induced meiotic resumption of cumulus cell-enclosed porcine oocytes via PKC pathway. Mol Cell Endocrinol (2006) 249(1-2):64–70. doi: 10.1016/j.mce.2006.01.008 16500744

[35] Wang C Xie H Song X Ning G Yan J Chen X. Lanosterol 14alpha-demethylase expression in the mouse ovary and its participation in cumulus-enclosed oocyte spontaneous meiotic maturation in *vitro* . Theriogenology (2006) 66(5):1156–64. doi: 10.1016/j.theriogenology.2006.01.065 16650467

[36] Wang C Xu B Zhou B Zhang C Yang J Ouyang H. Reducing CYP51 inhibits follicle-stimulating hormone induced resumption of mouse oocyte meiosis in *vitro* . J Lipid Res (2009) 50(11):2164–72. doi: 10.1194/jlr.M800533-JLR200 PMC2759822 19433477

[37] Scott RR Miller WL. Genetic and clinical features of p450 oxidoreductase deficiency. Horm Res (2008) 69(5):266–75. doi: 10.1159/000114857 18259105

[38] Fukami M Homma K Hasegawa T Ogata T. Backdoor pathway for dihydrotestosterone biosynthesis: implications for normal and abnormal human sex development. Dev Dyn (2013) 242(4):320–9. doi: 10.1002/dvdy.23892 23073980

[39] Flück CE Parween S Rojas Velazquez MN Pandey AV. Inhibition of placental CYP19A1 activity remains as a valid hypothesis for 46,XX virilization in P450 oxidoreductase deficiency. Proc Natl Acad Sci U.S.A. (2020) 117(26):14632–3. doi: 10.1073/pnas.2003154117 PMC7334497 32576700

[40] Krone N Hanley NA Arlt W. Age-specific changes in sex steroid biosynthesis and sex development. Best Pract Res Clin Endocrinol Metab (2007) 21(3):393–401. doi: 10.1016/j.beem.2007.06.001 17875487

[41] Reisch N Taylor AE Nogueira EF Asby DJ Dhir V Berry A. Alternative pathway androgen biosynthesis and human fetal female virilization. Proc Natl Acad Sci U.S.A. (2019) 116(44):22294–9. doi: 10.1073/pnas.1906623116 PMC6825302 31611378

[42] Parween S DiNardo G Baj F Zhang C Gilardi G Pandey AV. Differential effects of variations in human P450 oxidoreductase on the aromatase activity of CYP19A1 polymorphisms R264C and R264H. J Steroid Biochem Mol Biol (2020) 196:105507. doi: 10.1016/j.jsbmb.2019.105507 31669572

[43] Unal E Demiral M Yıldırım R Taş FF Ceylaner S Özbek MN. Cytochrome P450 oxidoreductase deficiency caused by a novel mutation in the POR gene in two siblings: case report and literature review. Hormones (Athens) (2021) 20(2):293–8. doi: 10.1007/s42000-020-00249-z 33123976

[44] Keber R Motaln H Wagner KD Debeljak N Rassoulzadegan M Ačimovič J. Mouse knockout of the cholesterogenic cytochrome P450 lanosterol 14alpha-demethylase (Cyp51) resembles Antley-Bixler syndrome. J Biol Chem (2011) 286(33):29086–97. doi: 10.1074/jbc.M111.253245 PMC3190716 21705796

[45] Laue K Pogoda HM Daniel PB van Haeringen A Alanay Y von Ameln S. Craniosynostosis and multiple skeletal anoMalies in humans and zebrafish result from a defect in the localized degradation of retinoic acid. Am J Hum Genet (2011) 89(5):595–606. doi: 10.1016/j.ajhg.2011.09.015 22019272 PMC3213388

[46] Ribes V Otto DM Dickmann L Schmidt K Schuhbaur B Henderson C. Rescue of cytochrome P450 oxidoreductase (Por) mouse mutants reveals functions in vasculogenesis, brain and limb patterning linked to retinoic acid homeostasis. Dev Biol (2007) 303(1):66–81. doi: 10.1016/j.ydbio.2006.10.032 17126317

[47] Ono K Sandell LL Trainor PA Wu DK. Retinoic acid synthesis and autoregulation mediate zonal patterning of vestibular organs and inner ear morphogenesis. Development (2020) 147(15):dev192070. doi: 10.1242/dev.192070 32665247 PMC7420839

[48] Pan P Zheng L Chen X Huang J Yang D Li Y. Successful live birth in a Chinese wOman with P450 oxidoreductase deficiency through frozen-thawed embryo transfer: a case report with review of the literature. J Ovarian Res (2021) 14(1):22. doi: 10.1186/s13048-021-00778-0 33526062 PMC7852152

[49] Zhang T Li Z Ren X Huang B Zhu G Yang W. Clinical and genetic analysis of cytochrome P450 oxidoreductase (POR) deficiency in a female and the analysis of a novel POR intron mutation causing alternative mRNA splicing : Overall analysis of a female with POR deficiency. J Assist Reprod Genet (2020) 37(10):2503–11. doi: 10.1007/s10815-020-01899-z PMC7550433 32725309

[50] Dewailly D Robin G Peigne M Decanter C Pigny P Catteau-Jonard S. Interactions between androgens, FSH, anti-Müllerian hormone and estradiol during folliculogenesis in the human normal and polycystic ovary. Hum Reprod Update (2016) 22(6):709–24. doi: 10.1093/humupd/dmw027 27566840

[51] Hamad H Ghazle and Patricia M. Newcomb. Sonographic diagnosis of antley-bixler PORD-type syndrome. J Diagn Med Sonography (2014) 2:93–8. doi: 10.1177/8756479314549583

[52] Krone N Reisch N Idkowiak J Dhir V Ivison HE Hughes BA. Genotype-phenotype analysis in congenital adrenal hyperplasia due to P450 oxidoreductase deficiency. J Clin Endocrinol Metab (2012) 97(2):E257–67. doi: 10.1210/jc.2011-0640 PMC3380101 22162478

[53] Guaragna-Filho G Castro CC Carvalho RR Coeli FB Ferraz LF Petroli RJ. 46,XX DSD and Antley-Bixler syndrome due to novel mutations in the cytochrome P450 oxidoreductase gene. Arq Bras Endocrinol Metabol (2012) 56(8):578–85. doi: 10.1590/s0004-27302012000800020 23295302

[54] Fan L Ren X Song Y Su C Fu J Gong C. Novel phenotypes and genotypes in Antley-Bixler syndrome caused by cytochrome P450 oxidoreductase deficiency: based on the first cohort of Chinese children. Orphanet J Rare Dis (2019) 14(1):299. doi: 10.1186/s13023-019-1283-2 31888681 PMC6937861

[55] Huang N Pandey AV Agrawal V Reardon W Lapunzina PD Mowat D. Diversity and function of mutations in p450 oxidoreductase in patients with Antley-Bixler syndrome and disordered steroidogenesis. Am J Hum Genet (2005) 76(5):729–49. doi: 10.1086/429417 PMC1199364 15793702

[56] Fukami M Nishimura G Homma K Nagai T Hanaki K Uematsu A. Cytochrome P450 oxidoreductase deficiency: identification and characterization of biallelic mutations and genotype-phenotype correlations in 35 Japanese patients. J Clin Endocrinol Metab (2009) 94(5):1723–31. doi: 10.1210/jc.2008-2816 19258400

[57] Fukami M Hasegawa T Horikawa R Ohashi T Nishimura G Homma K. Cytochrome P450 oxidoreductase deficiency in three patients initially regarded as having 21-hydroxylase deficiency and/or aromatase deficiency: diagnostic value of urine steroid hormone analysis. Pediatr Res (2006) 59(2):276–80. doi: 10.1203/01.pdr.0000195825.31504.28 16439592

[58] Iijima S Ohishi A Ohzeki T. Cytochrome P450 oxidoreductase deficiency with Antley-Bixler syndrome: steroidogenic capacities. J Pediatr Endocrinol Metab (2009) 22(5):469–75. doi: 10.1515/jpem.2009.22.5.469 19618668

[59] Charmandari E Nicolaides NC Chrousos GP. Adrenal insufficiency. Lancet (2014) 383(9935):2152–67. doi: 10.1016/S0140-6736(13)61684-0 24503135

[60] Auchus RJ Chang AY. 46,XX DSD: the masculinised female. Best Pract Res Clin Endocrinol Metab (2010) 24(2):219–42. doi: 10.1016/j.beem.2009.11.001 20541149

[61] Song T Wang B Chen H Zhu J Sun H. *In vitro* fertilization-frozen embryo transfer in a patient with cytochrome P450 oxidoreductase deficiency: a case report. Gynecol Endocrinol (2018) 34(5):385–8. doi: 10.1080/09513590.2017.1393663 29069987

[62] Homma K Hasegawa T Nagai T Adachi M Horikawa R Fujiwara I. Urine steroid hormone profile analysis in cytochrome P450 oxidoreductase deficiency: implication for the backdoor pathway to dihydrotestosterone. J Clin Endocrinol Metab (2006) 91(7):2643–9. doi: 10.1210/jc.2005-2460 16608896

[63] Pandey AV Kempná P Hofer G Mullis PE Flück CE. Modulation of human CYP19A1 activity by mutant NADPH P450 oxidoreductase. Mol Endocrinol (2007) 21(10):2579–95. doi: 10.1210/me.2007-0245 17595315

[64] Pandey AV Sproll P. Pharmacogenomics of human P450 oxidoreductase. Front Pharmacol (2014) 5:103. doi: 10.3389/fphar.2014.00103 24847272 PMC4023047

[65] Riddick DS Ding X Wolf CR Porter TD Pandey AV Zhang QY. NADPH-cytochrome P450 oxidoreductase: roles in physiology, pharmacology, and toxicology. Drug Metab Dispos (2013) 41(1):12–23. doi: 10.1124/dmd.112.048991 23086197 PMC3533425

[66] Flück CE Pandey AV. Impact on CYP19A1 activity by mutations in NADPH cytochrome P450 oxidoreductase. J Steroid Biochem Mol Biol (2017) 165(Pt A):64–70. doi: 10.1016/j.jsbmb.2016.03.031 27032764

[67] Shen AL O'Leary KA Kasper CB. Association of multiple developmental defects and embryonic lethality with loss of microsomal NADPH-cytochrome P450 oxidoreductase. J Biol Chem (2002) 277(8):6536–41. doi: 10.1074/jbc.M111408200 11742006

[68] Otto DM Henderson CJ Carrie D Davey M Gundersen TE Blomhoff R. Identification of novel roles of the cytochrome p450 system in early embryogenesis: effects on vasculogenesis and retinoic Acid homeostasis. Mol Cell Biol (2003) 23(17):6103–16. doi: 10.1128/MCB.23.17.6103-6116.2003 PMC180925 12917333

[69] Tee MK Huang N Damm I Miller WL. Transcriptional regulation of human P450 oxidoreductase: identification of transcription factors and influence of promoter polymorphisms. Mol Endocrinol (2011) 25:715–31. doi: 10.1210/me.2010-0236 PMC3082333 21393444

[70] Chun K Siegel-Bartelt J Chitayat D Phillips J Ray PN. FGFR2 mutation associated with clinical manifestations consistent with Antley-Bixler syndrome. Am J Med Genet (1998) 77(3):219–24. doi: 10.1002/(sici)1096-8628(19980518)77:3<219::aid-ajmg6>3.0.co;2-k 9605588

[71] Tsai FJ Wu JY Yang CF Tsai CH. Further evidence that fibroblast growth factor receptor 2 mutations cause Antley-Bixler syndrome. Acta Paediatr (2001) 90(5):595–7. doi: 10.1111/j.1651-2227.2001.tb00811.x 11430730

[72] Kamenický P Blanchard A Lamaziere A Piedvache C Donadille B Duranteau L. Cortisol and aldosterone responses to hypoglycemia and na depletion in women with non-classic 21-hydroxylase deficiency. J Clin Endocrinol Metab (2020) 105(1):dgz005. doi: 10.1210/clinem/dgz005 31529070

[73] Koyama Y Homma K Fukami M Miwa M Ikeda K Ogata T. Classic and non-classic 21-hydroxylase deficiency can be discriminated from P450 oxidoreductase deficiency in Japanese infants by urinary steroid metabolites. Clin Pediatr Endocrinol (2016) 25(2):37–44. doi: 10.1297/cpe.25.37 27212795 PMC4860514

[74] Dewailly D Lujan ME Carmina E Cedars MI Laven J Norman RJ. Definition and significance of polycystic ovarian morphology: a task force report from the Androgen Excess and Polycystic Ovary Syndrome Society. Hum Reprod Update (2014) 20(3):334–52. doi: 10.1093/humupd/dmt061 24345633

[75] Rotterdam ESHRE/ASRM-Sponsored PCOS consensus workshop group. Revised 2003 consensus on diagnostic criteria and long-term health risks related to polycystic ovary syndrome (PCOS). Hum Reprod (2004) 19(1):41–7. doi: 10.1093/humrep/deh098 14688154

[76] McCartney C Marshall JC. Polycystic ovary syndrome. N Engl J Med (2016) 375(14):1398–9. doi: 10.1056/NEJMc1610000 27705264

[77] Scott RR Gomes LG Huang N Van Vliet G Miller WL. Apparent manifesting heterozygosity in P450 oxidoreductase deficiency and its effect on coexisting 21-hydroxylase deficiency. J Clin Endocrinol Metab (2007) 92(6):2318–22. doi: 10.1210/jc.2006-2345 17389698

[78] Parween S Fernández-Cancio M Benito-Sanz S Camats N Rojas Velazquez MN López-Siguero JP. Molecular basis of CYP19A1 deficiency in a 46,XX patient with R550W mutation in POR: expanding the PORD phenotype. J Clin Endocrinol Metab (2020) 105(4):dgaa076. doi: 10.1210/clinem/dgaa076 32060549

[79] Williamson L Arlt W Shackleton C Kelley RI Braddock SR. Linking Antley-Bixler syndrome and congenital adrenal hyperplasia: a novel case of P450 oxidoreductase deficiency. Am J Med Genet A (2006) 140A(17):1797–803. doi: 10.1002/ajmg.a.31385 16906539

[80] Nakanishi K Yamashita A Miyamoto T Takeguchi R Furuya A Matsuo K. P450 oxidoreductase deficiency with maternal virilization during pregnancy. Clin Exp Obstet Gynecol (2016) 43(6):902–4. doi: 10.12891/ceog3172.2016 29944250

[81] Zhu WJ Cheng T Zhu H Han B Fan MX Gu T. Aromatase deficiency: a novel compound heterozygous mutation identified in a Chinese girl with severe phenotype and obvious maternal virilization. Mol Cell Endocrinol (2016) 15:433. doi: 10.1016/j.mce.2016.05.025 27256151

[82] Shackleton C Marcos J Arlt W Hauffa BP. Prenatal diagnosis of P450 oxidoreductase deficiency (ORD): a disorder causing low pregnancy estriol, maternal and fetal virilization, and the Antley-Bixler syndrome phenotype. Am J Med Genet A (2004) 129A(2):105–12. doi: 10.1002/ajmg.a.30171 15316970

[83] Metcalfe SA. Genetic counselling, patient education, and informed decision-making in the genomic era. Semin Fetal Neonatal Med (2018) 23(2):142–9. doi: 10.1016/j.siny.2017.11.010 29233487

[84] Hayward J Chitty LS. Beyond screening for chromosomal abnorMalities: Advances in non-invasive diagnosis of single gene disorders and fetal exome sequencing. Semin Fetal Neonatal Med (2018) 23(2):94–101. doi: 10.1016/j.siny.2017.12.002 29305293

[85] But WM Lo IF Shek CC Tse WY Lam ST. Ambiguous genitalia, impaired steroidogenesis, and Antley-Bixler syndrome in a patient with P450 oxidoreductase deficiency. Hong Kong Med J (2010) 16(1):59–62. 20124576

[86] Husebye ES Pearce SH Krone NP Kämpe O. Adrenal insufficiency. Lancet (2021) 397(10274):613–29. doi: 10.1016/S0140-6736(21)00136-7 33484633

[87] Bancos I Hahner S Tomlinson J Arlt W. Diagnosis and management of adrenal insufficiency. Lancet Diabetes Endocrinol (2015) 3(3):216–26. doi: 10.1016/S2213-8587(14)70142-1 25098712

[88] Onuki T Ohtsu Y Hiroshima S Sawano K Nagasaki K. Two cases of cytochrome P450 oxidoreductase deficiency with severe scoliosis and surgery requirement. Congenit Anom (Kyoto) (2021) 61(5):202–3. doi: 10.1111/cga.12434 34155696

[89] Idkowiak J Cragun D Hopkin RJ Arlt W. Cytochrome P450 Oxidoreductase Deficiency. In: Adam MP Everman DB Mirzaa GM Pagon RA Wallace SE Bean LJH Gripp KW Amemiya A, editors. . GeneReviews®. Seattle (WA: University of Washington, Seattle (2005). p. 1993–2023. 20301592

[90] Adolphs N Klein M Haberl EJ Graul-Neumann L Menneking H Hoffmeister B. Antley-Bixler-syndrome–staged management of craniofacial malformations from birth to adolescence–a case report. J Craniomaxillofac Surg (2011) 39(7):487–95. doi: 10.1016/j.jcms.2010.10.026 21146417

[91] Nordenskjöld A Holmdahl G Frisén L Falhammar H Filipsson H Thorén M. Type of mutation and surgical procedure affect long-term quality of life for women with congenital adrenal hyperplasia. J Clin Endocrinol Metab (2008) 93(2):380–6. doi: 10.1210/jc.2007-0556 18029470

[92] Gençay I Vargel I Büyükkoçak U Yazc I Apan A. Anesthetic risks associated with Antley-Bixler syndrome. J Craniofac Surg (2013) 24(1):e21–3. doi: 10.1097/SCS.0b013e318267be0f 23348324

[93] Flück CE Mullis PE Pandey AV. Reduction in hepatic drug metabolizing CYP3A4 activities caused by P450 oxidoreductase mutations identified in patients with disordered steroid metabolism. Biochem Biophys Res Commun (2010) 401(1):149–53. doi: 10.1016/j.bbrc.2010.09.035 20849814

[94] Merke DP Poppas DP. Management of adolescents with congenital adrenal hyperplasia. Lancet Diabetes Endocrinol (2013) 1(4):341–52. doi: 10.1016/S2213-8587(13)70138-4 PMC4163910 24622419

[95] Wang C Tian Q. The investigation of quality of life in 87 Chinese patients with disorders of sex development. BioMed Res Int (2015) 2015:342420. doi: 10.1155/2015/342420 26075230 PMC4449867

[96] Flück CE Miller WL. P450 oxidoreductase deficiency: a new form of congenital adrenal hyperplasia. Curr Opin Pediatr (2006) 18(4):435–41. doi: 10.1097/01.mop.0000236395.71956.5c 16915000

