# Supplementary material for: Diagnostic challenges and management advances in cytochrome P450 oxidoreductase deficiency, a rare form of congenital adrenal hyperplasia, with 46, XX karyotype
Source: Front Endocrinol (Lausanne). 2023 Aug 11;14:1226387. doi: 10.3389/fendo.2023.1226387 (PMC10453803; doi:10.3389/fendo.2023.1226387)
Supplement: Supplementary file 1 [file Table_1.docx]

Suspicion of PORD and associated assessment should be performed in the following conditions:

1.Maternal virilization during pregnancy, low maternal uE3 and obvious skeletal malformations on ultrasonography.

2.Multiple congenital abnormalities including ABS-like phenotype such as craniosynostosis and radiohumeral synostosis, as well as ambiguous genitalia, eg: clitoromegaly and labial fusion.

3.In phenotypically normal individuals with primary amenorrhea and oligomenorrhea and elevated P, PORD should also be suspected, which is easily misdiagnosed.

4.Gene test of POR is recommended to confirm diagnosis and mandatory in non conclusive cases.

5.Chromosomal test is required to help diagnosis and consequent sex assignment.

6.ACTH stimulation test is needed to evaluate the presence and severity of adrenal insufficiency which is readily overlooked.

**Key points in management of PORD:**

1.Glucocorticoid replacement is often required to treat potential adrenal insufficiency and prevent adrenal crisis, with regular administration or stress dose cover.

2.Sex steroid supplement therapy is essential to promote pubertal development and decrease ovarian cysts, sometimes combined with GnRH analogue.

3.Proper sex assignment is important for patients with ambiguous genitalia.

4.Multiple interdisciplinary operations may be needed for correction of congenital developmental malformations, such as craniosynostosis.

5.Successful pregnancy outcomes can be achieved by IVF-FET following adequate control of hormonal imbalances, albeit no spontaneous pregnancy has occurred in PORD.

6.Reinforced education and psychosocial support may be helpful to improve the clinical outcomes.

Table 1: The diagnosis and evaluation of POR Table 2: Several key points in management of PORD.
